# Changes in Allergy Symptoms and Depression Scores Are Positively Correlated In Patients With Recurrent Mood Disorders Exposed to Seasonal Peaks in Aeroallergens

**DOI:** 10.1100/tsw.2007.286

**Published:** 2007-12-17

**Authors:** Teodor T. Postolache, Manana Lapidus, Evan R. Sander, Patricia Langenberg, Robert G. Hamilton, Joseph J. Soriano, Jessica S. McDonald, Nancy Furst, Jie Bai, Debra A. Scrandis, Johanna A. Cabassa, John W. Stiller, Theodora Balis, Alvaro Guzman, Alkis Togias, Leonardo H. Tonelli

**Affiliations:** ^1^ Mood and Anxiety Program, Department of Psychiatry, University of Maryland School of Medicine, Baltimore, MD, USA; ^2^ Epidemiology and Preventive Medicine, University of Maryland School of Medicine, Baltimore, MD, USA; ^3^ Division of Allergy and Clinical Immunology, Department of Medicine, Johns Hopkins University School of Medicine, Baltimore, MD, USA; ^4^ National Center for the Treatment of Phobias, Anxiety and Depression, Washington DC, USA; ^5^ Neurology Consultation Service, St. Elizabeth's Hospital, Washington, DC, USA; ^6^ Pulmonary and Critical Care Medicine, Johns Hopkins Asthma and Allergy Center, Baltimore, MD, USA; ^7^ Behavioral Neuroimmunology, Mood and Anxiety Program, Department of Psychiatry, University of Maryland School of Medicine, Baltimore, MD, USA

**Keywords:** allergy, aeroallergen, tree pollen, allergen specific IgE, major depression, bipolar disorder

## Abstract

Although growing evidence supports an association between allergy, allergens and depression, it remains unknown if this relationship is between “states” (possible triggers) or “traits” (possible vulnerabilities). We hypothesized that patients with recurrent mood disorders who are sensitized to tree pollen (as determined by allergen specific IgE antibodies), in comparison to those who are not sensitized, would report larger negative changes in mood during exposure to tree pollen in spring. We also hypothesized that differences between high and low tree pollen periods in self reported allergy symptoms would correlate positively with differences in self reported depression scores. We present 1-year preliminary data on the first 51 patients with unipolar or bipolar disorder (age: 19-63 years, 65% female, twelve patients were tree-pollen IgE positive). Ratings of mood and allergic disease status were performed once during the peak airborne pollen counts and once during the period of low airborne pollen counts, as reported by two local pollen counting stations. Linear regression models were developed to examine associations of changes in depression scores (dependent variable) with tree pollen sensitization, changes in the allergy symptom severity score, adjusted for gender and order of testing. We did not confirm the hypothesized relationship between a specific tree pollen sensitization and changes in mood during tree pollen exposure. We did confirm the hypothesized positive relationship between the changes in allergy symptoms and changes in subjects' depression scores (adjusted p<0.05). This result is consistent with previous epidemiological evidence connecting allergy with depression, as well as our recent reports of increased expression of cytokines in the prefrontal cortex in victims of suicide and in experimental animals sensitized and exposed to tree pollen. A relationship between *changes* in allergy symptom scores and *changes* in depression scores supports a state-level rather than only trait-level relationship, and thus lends optimism to future causality-testing interventional studies, which might then lead to novel preventative environmental interventions in mood disorders.

## INTRODUCTION

Major depression and bipolar disorder represent key public health issues due to their substantial prevalence, recurrence, and devastating effects in middle adulthood[1]. In addition, the great majority of those who commit suicide are depressed at the time of death, and certain features and associations of depression represent important risk factors for suicide[2].

Allergic disease is also widespread with various manifestations affecting over 50% of the population[3]. In particular, allergic rhinoconjunctivitis is a common disorder with perennial and seasonal variants, as illustrated by peaks in rhinitis during periods of maximal tree pollen exposure[4,5,6]. Between 10% and 30% of people have allergic rhinoconjunctivitis[5], exceeding the prevalence of Major Depression by at least two-fold.

There is increasing evidence for an association between depression and allergy[7,8,9,10]. In addition, we reported that mood worsening occurs when atmospheric pollen is high and it is associated with a greater seasonality of mood and with seasonal affective disorder (SAD) of non-winter type[11]. Cohort studies have found a greater association between depression and allergy in women as compared to men[12,13]. However, it is unknown if this association is true or spurious, and if true, if it represents trait (vulnerability)- level relationship or if mood-states are involved, i.e. if there is adequate evidence to suggest that exposure to allergens might trigger mood changes in vulnerable individuals. Thus, a study relating *change* in allergen exposure, to *changes* in allergy scores, and to *changes* in depression scores, has no precedent to our knowledge, and would be important to direct our future investigation of a possible allergy-depression link.

One of the most highly replicated findings in psychiatric epidemiologic research is the seasonal spring peak in suicide, coinciding with the peak of depression decompensation in the spring[14-20]. There is also a less replicated and less marked suicide peak in the fall[19-23]. These peaks of mood decompensation and suicide[24,25] correspond to peaks of seasonal allergen exposure. Specifically, tree pollen has its peak pollen season in late spring, while airborne ragweed peaks in early fall. Allergies to aeroallergens trigger a series of cellular and molecular processes that start in the upper airways. The initial reaction to the allergen involves cross-linking of allergen-specific IgE by exposure to aeroallergens that results in mast cell activation and the release of inflammatory mediators. Among these inflammatory mediators are cytokines predominantly involved in antiparasitic immunity (“TH2 response”) such as Il-4, Il-5 and Il-13, and cytokines predominantly involved in the antiviral/antibacterial immunity (“TH1 response”). Increases in activity of TH1 cytokines, such as TNF-α have been shown to trigger depressive-like behaviors in experimental animal models [26-30] as well as decompensation of depression in humans[31]. There have been instances in which cytokine-treated patients with chronic hepatitis or cancer have experienced an increase in depressive symptoms, including suicidal ideation and attempted suicide[32-34]. Even a low concentration of the cytokine-promoting endotoxin (lipopolysaccharide) below the dose necessary to generate a “sickness behavior” can trigger depressive symptoms and anxiety[35]. It has been further suggested that cytokines from the periphery may stimulate the production of other cytokines thereby promoting inflammatory responses in the brain[36].

We have also conceptualized that allergy may be viewed as a misdirected antiparasitic response with TH2 cytokines (Il-4, Il-5, Il-13) affecting brain function and contributing to a potential relationship between allergy, depression and suicide[37]. Similarly, our group reported for the first time to our knowledge, that expression of TH2 cytokines occurs in the human brain and appears to be increased in victims of suicide as compared to controls who died from other causes [38].

In a preliminary study[39], we reported that the relative rate of suicide in younger women increased two-fold during the peak tree pollen season as compared to the low tree pollen season. The objective of the current study was to investigate whether spring worsening of mood disorders is related to seasonal allergies. Specifically, we hypothesized that: (a) IgE anti-tree pollen positive patients, as compared to patients negative for allergen-specific IgE antibody, will have a greater increase in depression scores during the peak airborne tree pollen interval versus the low tree pollen interval, and (b) changes in depression scores between low vs. peak pollen intervals in spring will be positively correlated with changes in allergy symptom scores between the same interval.

## METHODS

The Institutional Review Board of the University of Maryland School of Medicine approved the study. After a full explanation of the study, each participant signed both a screening consent and consent to participate in the protocol. The capacity of participants to sign the consents was evaluated using a semi-structured interview.

### Screening

All subjects were initially prescreened by phone with a questionnaire to rule out those reporting any major medical illness (e.g., cancer, hepatitis or HIV infection), a previous diagnosis of a psychotic disorder, winter-type seasonal affective disorder, illicit drug or alcohol dependence and pregnancy or an intention to become pregnant during the course of the study. At the first visit following screening consent, 6 cc of whole blood were drawn and serum was isolated. Subjects were included or excluded on the basis of a multi-allergen serological test (Phadiatop, ImmunoCAP 250, Phadia, Uppsala, Sweden), a single, qualitative multi-allergen screening assay for IgE antibody specific for a spectrum of indoor and outdoor aeroallergens that induce aeroallergen-related allergic disease. To determine each subject's sensitization to spring and/or fall pollen allergens, serum from each subject was reanalyzed for IgE antibody to individual allergens using the ImmunoCAP 250. Quantitative levels of IgE antibody specific for ash, beech, birch, elm, maple, poplar, sycamore, oak and ragweed pollen allergens were reported in kUa/L using 0.35 kUa/L as the positive threshold criterion[40].

Subjects were interviewed using the mood disorders, substance and psychotic disorders modules of the Structured Clinical Interview for DSM-IV (SCID)[41]. Individuals were included in the study if they had unipolar or bipolar depressive disorder and did not have any active substance abuse/dependence or psychotic disorder. For medications currently taken, patients were excluded if they were using intranasal corticosteroids or montelukast, but they were included if they were using antihistamine or decongestant medications. This decision was based on our hypothesis that inflammatory mediators and blood cytokine levels are not affected by the latter as compared to the former medications.

Group assignment was based on the subject's IgE serology results. All tree pollen IgE positive subjects were included in the study as the experimental group. Only IgE antibody negative subjects were included in the control group. Those individuals with a positive multi-allergen screen (Phadiatop) but who were negative for tree pollen specific IgE antibodies were excluded and referred to an allergist for appropriate follow-up.

## Mood Ratings and Allergic Disease Assessment

Ratings of mood and allergic disease status were performed once during the peak pollen season and once during the period of low airborne pollen counts. Depression scores for the week preceding the interview were obtained using the Structured Interview Guide for the Hamilton Depression Rating Scale – Seasonal Affective Disorder Version (SIGH-SAD)[42]. Hypomanic/manic scores were obtained using the Hypomania Interview Guide (including hyperthermia) for Seasonal Affective Disorder (HIGH-SAD)[43]. The Allergy Symptom Severity Assessment (ASSA)[44] enabled participants to self-report the severity of their allergic symptoms. This questionnaire consists of five structured Likert scales where responses included: (a) sneezing, (b) rhinorrhea, (c) itchy nose, palate or throat, (d) itchy, watery or red eyes, and (e) congestion. Possible answers were graded on a scale from 0 to 4: 0 = no symptoms, 1 = mild (no discomfort), 2 = moderate (discomfort but no interference with functioning), 3 = severe (interference with normal functioning), and 4 = very severe (requiring medical attention). The evaluators and participants were masked to the specific IgE antibody results.

## Pollen Counts and Pollen Intervals

Data on airborne pollen and fungal spore concentrations was obtained from two local pollen-counting stations that were enrolled in the Aeroallergen Monitoring Network/American Academy of Allergy Asthma and Immunology. Each site had credentialed pollen counters who identified and quantified pollen levels morphologically from air samples. Pollen concentrations are expressed as the daily average number of pollen grains per cubic meter of air for the different pollen groups. Pollen data from counting stations in Washington, DC and Baltimore were emailed to the study office daily from the American Academy of Allergy, Asthma, and Immunology (AAAAI). Starting in February, the data from these stations were recorded daily on the AAAAI website www.aaaai.org and confirmed by a research assistant who entered the data into a computerized database. The data were used to relate the genus of tree pollen counts to the specificity of the IgE in the blood of each study subject.

The periods of high or low levels of atmospheric tree pollen were identified as after January 15 and before July 1$^{st}$ of 2007, respectively. The pre-pollen interval began on January 15 and ended when the subject's first tree pollen allergen count exceeded 10 grains/m$^3$. It has been previously reported that tree pollen sensitive individuals report allergic symptoms starting just below this level[45]. The cut-off pollen count value to identify a peak tree pollen interval was $\geq$ 90 grains/m$^3$. For some participants, their low pollen period visit occurred during the pre-peak pollen season, and for others it occurred following the peak pollen period. This actual time of the visit was designed to be one of convenience, which enabled the subjects to participate when they were able to participate, rather than as a result of a randomized defined date.

## Statistical Methods

Initial descriptive analyses included examining distributions of all variables for outliers and extreme skewness. The delta variables for depression, anxiety, insomnia, and symptom severity were computed as the difference between the levels obtained at the high versus low pollen period associated visits. The mean and standard deviation of continuous variables and proportions were computed for categorical variables such as the IgE anti-tree pollen status. Proportions were compared by Pearson chi-square tests, and means by t-tests.

Linear regression models were developed to examine potential associations between high/low pollen changes in the allergy symptom severity score and changes in SIGH-SAD depression (total, typical, atypical, and insomnia scales), adjusted for important covariables. Variables were included in the regression models if they were statistically associated with an outcome, their inclusion changed the observed association of symptom score, or they were believed to be important based on the depression and allergy literature (e.g., gender). The variables that did not change the associations of symptom scores with outcomes were removed from the models. Because some low pollen visits occurred before or after the high pollen period, the "order of visit" variable was included in all models.

**Table 1.**
**Characteristics of Study Patients Combined and By Tree Pollen Positivity**

| | **Total** N=51 | **Tree-pollen Positive** N=12 (24%) | **Tree-pollen Negative** N=39 (76%) | **p-value*** |
|---|---|---|---|---|
| Age, mean (SD) | 45.4 (11.1) | 41.8 (13.1) | 46.5 (10.4) | 0.20 |
| Gender male (%) | 35.3 | 41.7 | 33.3 | 0.60 |
| Race (%) | | | | |
| Caucasian | 76.5 | 66.7 | 79.5 | 0.47 |
| African-American | 21.6 | 33.3 | 18.0 | |
| Hispanic | 2.0 | 0.0 | 2.6 | |
| Allergy symptoms (%) | | | | |
| Low pollen | 80.0 | 81.8 | 79.5 | 0.86 |
| High pollen | 95.7 | 90.9 | 97.2 | 0.36 |
| Nasal symptoms present (%) | | | | |
| Low pollen | 56.0 | 63.6 | 53.9 | 0.56 |
| High pollen | 63.8 | 72.7 | 61.1 | 0.48 |
| Depression score, mean (SD) | | | | |
| Low pollen | 13.2 (10.3) | 13.1 (10.2) | 13.2 (10.4) | 0.97 |
| High pollen | 14.1 (10.7) | 16.1 (11.9) | 13.4 (10.4) | 0.48 |
| Diagnosis (%) | | | | |
| Major depression | 68.6 | 66.7 | 69.2 | 0.87 |
| Bipolar disorder | 31.4 | 33.3 | 30.8 | |
| Medications (%) | | | | |
| Antidepressant | 86.3 | 75.0 | 89.7 | 0.19 |
| Mood stabilizer | 27.5 | 25.0 | 28.2 | 0.83 |
| Anxiolytic | 21.6 | 8.3 | 25.6 | 0.20 |
| Antipsychotic | 19.6 | 25.0 | 18.0 | 0.59 |
| Thyroid hormones | 17.7 | 8.3 | 20.5 | 0.34 |
| Sleep medication | 11.8 | 8.3 | 12.8 | 0.67 |
| Antihistaminic or decongestant | 7.8 | 16.7 | 5.1 | 0.19 |
| Low pollen visit before high pollen visit | 78.4 | 41.7 | 89.7 | 0.0004 |

*p-values from t-test comparing means and chi-square comparing proportions

## RESULTS

Table 1 presents the demographics of the sample ($n$ = 51). The sample was primarily female (65%). The race distribution included 11 African Americans, 39 Caucasians, and one Hispanic. The mean age was 44.8 years with a range of 19-63 years. Twelve participants (24%) were tree pollen positive based on their

IgE antibody serology. A majority of sensitized subjects experienced allergy symptoms at both low (80%) and high (96%) pollen periods. Nasal symptoms also afflicted more than half of our sample (low: 56% versus high: 64%). Their mean SIGH-SAD total depression scores ranged from 13.2 to 14.1 for low and high pollen periods, respectively, with considerable variability. There was no significant difference in depression scores observed between subjects who were sensitized to pollen (tree pollen specific IgE antibody positive) versus those who were Phadiatop negative for any of the variables evaluated except for the "order of visit" variable. 90% of subjects who were tree pollen specific IgE antibody negative made their low pollen visit before the high pollen period, while this was the case for only 42% of tree pollen sensitive subjects. Diagnostic distribution and medication levels are also presented in Table 1.

**Table 2.**
**Results of Multiple Linear Regression Analyses of Associations of Change in Allergy Symptom Scores With Changes in Depression and Sleep Scores**

| | Dependent Variable (High - Low Pollen Score) | | | | | | | | | | | |
|---|---|---|---|---|---|---|---|---|---|---|---|---|
| | Total SIGH-SAD | | | Typical SIGH-SAD | | | Atypical SIGH-SAD | | | Insomnia | | |
| | β | SE | p-value* | β | SE | p-value | β | SE | p-value | β | SE | p-value |
| **Allergy Symptom Score (high - low pollen)** | 1.03 | 0.46 | 0.03 | 0.67 | 0.30 | 0.03 | 0.36 | 0.21 | 0.10 | 0.13 | 0.10 | 0.23 |
| **Gender male** | 1.83 | 3.10 | 0.56 | -0.49 | 2.06 | 0.81 | 2.32 | 1.44 | 0.12 | -0.44 | 0.72 | 0.54 |
| **Tree Positive (Yes vs No)** | 4.91 | 4.13 | 0.24 | 3.07 | 2.74 | 0.27 | 1.84 | 1.92 | 0.34 | -0.32 | 0.94 | 0.73 |
| **Low pollen visit before high** | 8.41 | 4.12 | 0.05 | 6.22 | 2.73 | 0.03 | 2.19 | 1.92 | 0.26 | -0.69 | 0.94 | 0.47 |

Linear regression analyses examined associations between changes (low versus high pollen periods) in allergy symptom scores and SIGH-SAD depression and insomnia scores, adjusted for gender, tree pollen positivity, and order of low and high pollen visits (Table 2). Significant associations were observed between changes in the allergy symptom severity (ASSA) and changes in both total and typical depression scores. Specifically, a 1 unit increase in allergy symptom change was associated with an increase of 1.03 in the total SIGH-SAD change score ($p$=0.03) and 0.67 in the typical subscale ($p$=0.03). Symptom changes were not significantly associated with changes in the atypical SIGH-SAD items ($p$=0.10) or the insomnia subscale ($p$=0.23). Gender and tree pollen positivity were not significantly associated with any outcome, and removing them from models did not change any results. Patients whose low pollen visit preceded the high pollen visit had a significantly higher increase in both the total SIGH-SAD score (8.4) and the typical depressive symptoms subscale (6.2).

## DISCUSSION

We report a statistically significant positive relationship between the changes in upper respiratory allergy symptoms and changes in the subject's depression scores over the spring from the low to high pollen periods. The results of the present study are consistent with those of previous research that has indicated an association between allergic disease and depression decompensation[7,8,10-13,39,46]. This association is also consistent with our recent report that the induction of nasal inflammation results in depression-like behavior in animals[30], and our animal data showing an increased expression of TH2 cytokines in the brain with sensitization and exposure to tree pollen[47]. The data have not confirmed our working hypothesis that tree pollen specific IgE antibody positivity can predict the severity of mood worsening.

This finding may have resulted from the small number of tree pollen specific IgE positive individuals recruited at the time of this preliminary report. An alternate explanation might be represented by the unexpectedly large proportion of controls that developed allergic symptoms during the peak pollen period, despite being IgE anti-tree pollen negative. One can offer several speculative explanations for this observation. First, the nasal symptoms developed by subjects in the control group may reflect "non-allergic rhinitis", a syndrome of unclear etiology. Non-allergic rhinitis is characterized by subjective hyperresponsiveness of the nasal mucosa to non-allergenic environmental triggers including changes in weather, irritants, or pollutants[48]. Springtime is characterized by abrupt atmospheric changes, which may impact both nasal airways (sudden fluctuations in atmospheric pressure and temperature) and also airborne pollen counts (light, temperature, humidity, wind, etc). Second, it is possible that our control subjects could have been sensitive and exposed to other allergens that were in the air during the study, but not screened for by the Phadiatop test. Third, it has been recently suggested that some individuals, who develop nasal symptoms in the absence of serum IgE or a positive skin test to prevalent environmental allergen, may have a 'local form' of allergic disease involving IgE produced only in the nasal mucosa[49]. Finally, exogenous proteases from pollens and other aeroallergens, which act on specific molecules such as protease-activated receptors (PARs), may cause inflammation by mechanisms other than IgE activation of effector cells[50,51]. This is a variant of innate immunity but it is directed at multi-cellular parasites rather than unicellular organisms as would occur with the "classic" LPS-induced innate immune response[52]. Yet, there is so far no solid evidence that such a mechanism is operative in human nasal disease.

Among the limitations of the study is the qualitative nature of the multi-allergen screen for assigning sensitization to aeroallergens. Because it only detects specific IgE antibody to 15 common environmental indoor and outdoor aeroallergens, the study participant may have been sensitive to an allergen other than these 15 aeroallergens. The order of the visits was not random. The allergic symptom assessments were performed as a self-report and did not use more accurate biological markers of upper airway allergic disease such as measures of inflammation with mediator release in nasal lavage or peripheral blood. These analyses are planned for future reports. Station level pollen counts only suggest individual exposure, but do not measure it precisely, particularly in participants' work and home microclimates. A questionnaire to screen all the subjects for past history of allergic rhinitis was not used.

In evaluating the association between severity of allergy symptoms and mood, the potential subjectivity in the measurements needs to be taken into consideration. For instance, a reporting style consistent with neuroticism could elevate the self-reported allergy and depression symptoms, and thus create a spurious relationship between them. The role of neuroticism in this relationship was examined in a recent publication[53]. The authors identified an increased likelihood of allergic disease in individuals with major depression as well as in those with neuroticism as a personality trait. When accounting for gender, the relationship to allergic disease differed as well. In males, allergy was significantly associated with the presence of neuroticism but not with depression, while in the female group, allergic disease was strongly associated with depression but not to neuroticism. The study concluded that it is unlikely that neuroticism is associated with allergy-induced depression[53]. Because "neuroticism" is considered a trait rather than a psychological state, its effects are minimized by measuring individual *changes* in mood and allergy symptoms, rather than their *absolute values*.

Allergies can influence mood by several potential mechanisms. There may be somatic changes such as discomfort caused by allergic inflammatory processes in the upper airway, that may affect well being. This situation may lead to other possible mediators affecting mood, such the use of medications such as antihistamines or vasoconstrictors, or disturbance of sleep caused by multiple factors including obstruction of the airways[49,50]. The release of inflammatory mediators including cytokines is one likely mechanism that may promote the worsening in mood. This mood worsening can occur by either acting directly in the brain or through other pathways such as interactions with the HPA-axis and/or the IDO enzyme. Cytokines have been shown to induce depression and anxiety. Our findings relating allergic symptoms with depression scores may be explained in part by the release of cytokines during allergic reactions. *In vitro* studies have shown that certain human polymorphisms increase the expression of cytokines including TNF-α[51,52,54], IL-13[55], and IFN-γ[47]. In addition, certain cytokine polymorphisms increase susceptibility to allergic disease such as asthma[54,55]. Moreover, allergic symptoms have been correlated with the amount of cytokines released during some allergic reactions[55]. Thus it is possible that depression scores in some individuals may reflect the increased amount of cytokine release during allergic processes. This issue is a matter to be clarified with future investigations.

Despite its possible limitations, this study is the first to explore the relationship between sensitization to specific aeroallergens and the presence of depression decompensation during allergen exposure. In our previous manuscript[11], allergy symptoms and mood changes were recorded from memory. The current study examined the severity of allergy symptoms in relationship to concurrently monitored depression severity. Our current result supports a relationship at a "state" rather than "trait" (i.e. vulnerability) level, a more optimistic alternative, as it implies possible triggering, and prophylactic potential. Obviously, causality, and direction of causality are not implied by our results, and would require experimental/interventional studies to see if exposure to aeroallergens triggers exacerbation of mood disorders in vulnerable patients. If confirmed in larger studies, the implications of our findings could be considerable. Clinicians should be particularly observant of those patients with a history of depression during the peak tree pollen period in the spring, which is temporally associated with a significant spring peak in suicide[39]. Future studies should include replication of these findings and an examination of the mechanisms responsible for this reported relationship. Such studies may result in an informed search for novel pharmacological agents, and aeroallergen exposure reduction paradigms, which could reduce depression exacerbation with its concomitant suicide risk, related to recurrent mood disorders.

## ACKNOWLEDGEMENTS

Supported by grant number R21 MH075891-01A1 from the National Institute of Mental Health (Principal Investigator: Teodor T. Postolache), by research funds from the Johns Hopkins University Dermatology Allergy and Clinical Immunology (DACI) Reference Laboratory and by the University of Maryland General Clinical Research Center Grant M01 RR 16500, General Clinical Research Centers Program, National Center for Research Resources (NCRR), NIH. In addition, Dr. Postolache was supported by R01MH074891 from NIMH and by a NARSAD Independent Investigator Award. The content is solely the responsibility of the authors and does not necessarily represent the official views of the National Institute Of Mental Health or the National Institutes of Health. The authors thank Ms. Belzora Joppy and Dr. Bernard Vittone for their help with recruitment, and the staff of the GCRC/ University of Maryland for their important contribution to the execution of this study.
